# Supplementary material for: A Global Screen for Assembly State Changes of the Mitotic Proteome by SEC-SWATH-MS
Source: Cell Syst. 2020 Feb 26;10(2):133–155.e6. doi: 10.1016/j.cels.2020.01.001 (PMC7042714; doi:10.1016/j.cels.2020.01.001)

Q8WXW3 | PIBF1_HUMAN | PIBF1 C13orf24 PIBF
Monomer MW [kDa]: 89.805 Monomer expected elution fraction: 41

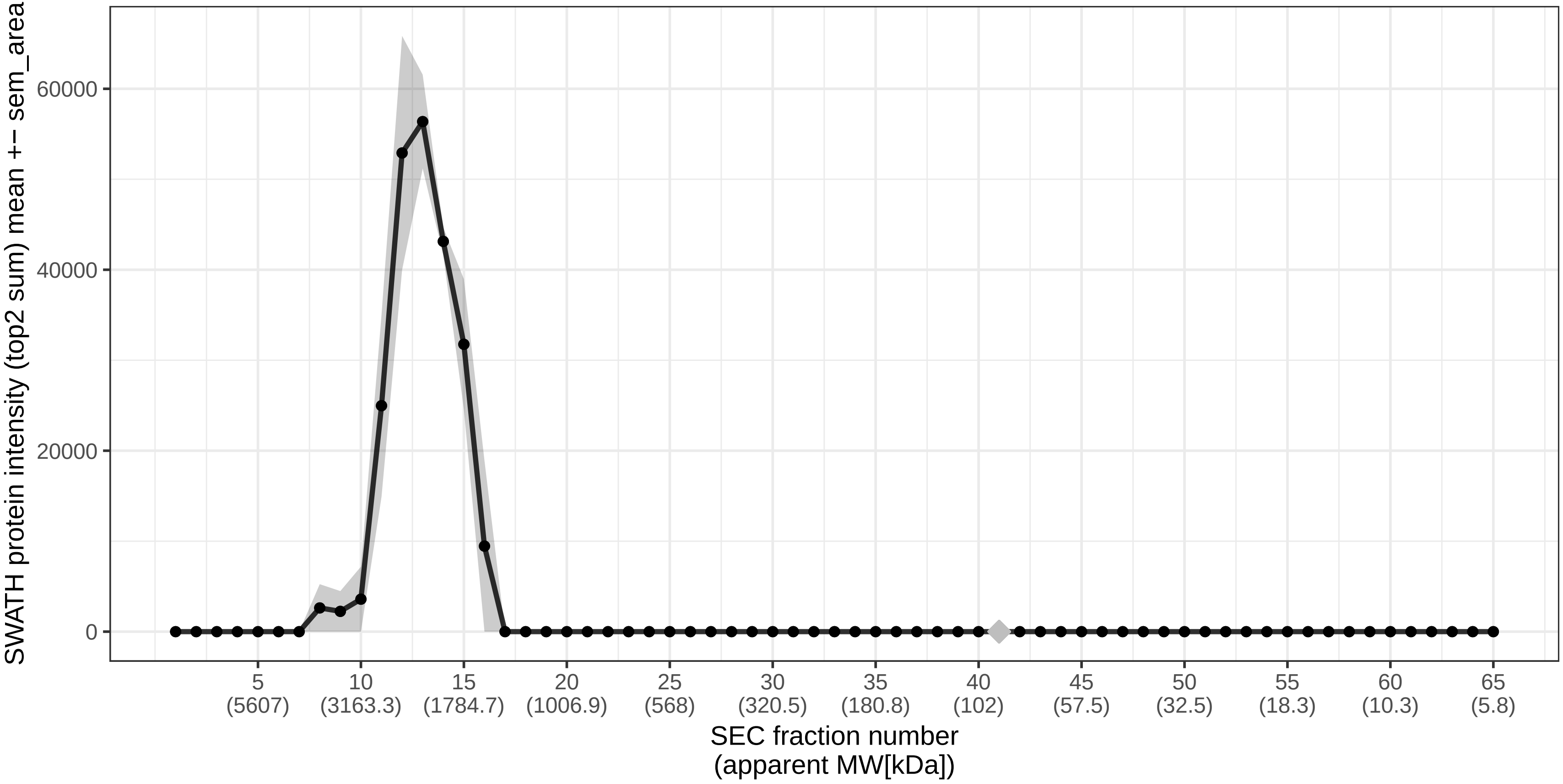

Supplement: Data S1. SEC-SWATH-MS Protein Chromatograms, Related to Figure 1 [file mmc6.zip › SECchrom_Q8WXW3_PIBF1_HUMAN_PIBF1_C13orf24_PIBF.pdf]
